# The effect of intravertebral anesthesia on bone cement implantation syndrome in aged patients: A single-center 5-year retrospective study

**DOI:** 10.1097/MD.0000000000004775

**Published:** 2016-09-09

**Authors:** Qian Chen, Chun Huang, Ya-Jun Zhang

**Affiliations:** Department of Anesthesiology, East branch, Sichuan Provincial People's Hospital, Sichuan Academy of Medical Science, Chengdu 610110, China.

**Keywords:** bone cement implantation syndrome, hemiarthroplasty, intravertebral anesthesia

## Abstract

The aim of the study was to assess the effect of commonly used intravertebral anesthesia on bone cement implantation syndrome (BCIS) in aged patients undergoing hemiarthroplasty.

The medical records of 1210 aged patients receiving hemiarthroplasty under intravertebral anesthesia were retrospectively reviewed. Anesthesia charts for all patients were reviewed for central venous pressure, mean arterial pressure, arterial oxygen saturation, and heart rate before, during, and after cementation. Each patient was classified into no BCIS (grade 0) or BCIS grade 1, 2, or 3 according to the degree of hypotension, arterial desaturation, or loss of consciousness around cementation. Changes in these grades after cementation were compared according to the ways of intravertebral anesthesia used.

Among all included patients, 72.2% (874/1210) showed grade 1 or higher grade of BCIS after cementation. Compared with spinal-epidural anesthesia, single epidural anesthesia showed adjusted odds ratios (95% confidence interval) of 1.25 (1.13–1.43) for grade 1, 1.36 (0.83–2.06) for grade 2, and 3.55 (1.52–7.06) for marked postoperatively grade 3 of BCIS versus grade 0 (Type III *P* *<* 0.0001).

Single epidural anesthesia was associated with increased odds for elevation of these grades after cementation compared with spinal-epidural anesthesia.

## 1 Introduction

Hemiarthroplasty is a routine surgical operation in the treatment of hip diseases of aged people such as hip arthritis, femoral head necrosis, and trauma.^[1,2]^ However, bone cement implantation syndrome (BCIS), which most often occurs in cemented hemiarthroplasty, is a well known and potentially fatal complication.^[3]^ The syndrome is characterized by hypoxia,^[4,5]^ sudden loss of arterial pressure,^[6]^ pulmonary hypertension,^[7]^ arrhythmias,^[8]^ loss of consciousness, and eventually cardiac arrest.^[9]^

Aged patients usually accompanied with pathological changes in respiratory and circulatory system; however, the effect of intravertebral anesthesia on BCIS in aged patients is unclear. Thus, it is particularly important to choose a reliable way of anesthesia to ensure the patient's safety and improve the success rate of surgery.

The severity classification of BCIS have been proposed recently:^[10]^ grade 1 was defined as a drop (> 20%) in systolic arterial pressure (SAP) or moderate hypoxia (arterial oxygen saturation < 94%), grade 2 as severe hypoxia (arterial oxygen saturation < 88%) or hypotension (a decrease in SAP > 40%) or unexpected loss of consciousness, and finally, grade 3 was defined as cardiovascular collapse requiring cardiopulmonary resuscitation.

The purpose of this study was to evaluate the effect of the commonly used spinal-epidural anesthesia and single epidural anesthesia, on the incidence of BCIS in cemented hemiarthroplasty for hip fractures, using this severity classification of BCIS.

## 2 Methods

The study was approved by the Ethics Committee of the Sichuan Academy of Medical Science and written informed consent was waived by the Ethics Committee because of the retrospective observational nature of the study. In this retrospective cohort study, the medical records of all consecutive patients underwent cemented hemiarthroplasty under spinal-epidural anesthesia and single epidural anesthesia for femoral neck fracture from January 1, 2010, to December 30, 2014, at Sichuan Provincial People's Hospital were screened for eligibility.

We collected data regarding anesthesia way, age, gender, American Society of Anesthesiologists (ASA) risk score, surgery time, and coexisting diseases including liver disease, renal impairment (serum creatinine > 150 μmol L^−1^), diabetes mellitus, previous stroke, and hypertension. However, coexisting dementia, arrhythmias, and chronic obstructive pulmonary disease (COPD) were excluded because of their potential effect on BCIS grade.

BCIS grade: The mean systolic pressure, arterial oxygen saturation, and heart rate were reviewed from anesthesia charts at 4 occasions (T1-T4): (T1) for a period of 10 to 15 minutes before intravertebral anesthesia was given, (T2) for a period of 10 to 15 minutes before implantation of bone cement, (T3) for a period of 10 to 15 minutes after implantation of bone cement, and (T4) on arrival to the post-anesthesia recovery unit. The lowest SAP recorded within 15 minutes after cementation was used to score the severity of BCIS. Each patient was classified to group BCIS grade 0, 1, 2, or 3 according to above principles.^[10]^

Statistical analysis was performed using SPSS statistical software (SPSS system for Windows, version 13.0). Patient characteristics and perioperative variables between BCIS grade groups were compared using the Kruskal–Wallis test for continuous variables and Pearson χ2 or Fisher exact test for categorical variables. Multinomial logistic regression was performed to evaluate risk factors associated with BCIS grade, and the adjusted odds ratio of each factor was calculated. The interaction terms between each variable in the model and ways of intravertebral anesthesia used were examined. Values of *P* < 0.05 were considered significant.

## 3 Results

**Figure 1:**
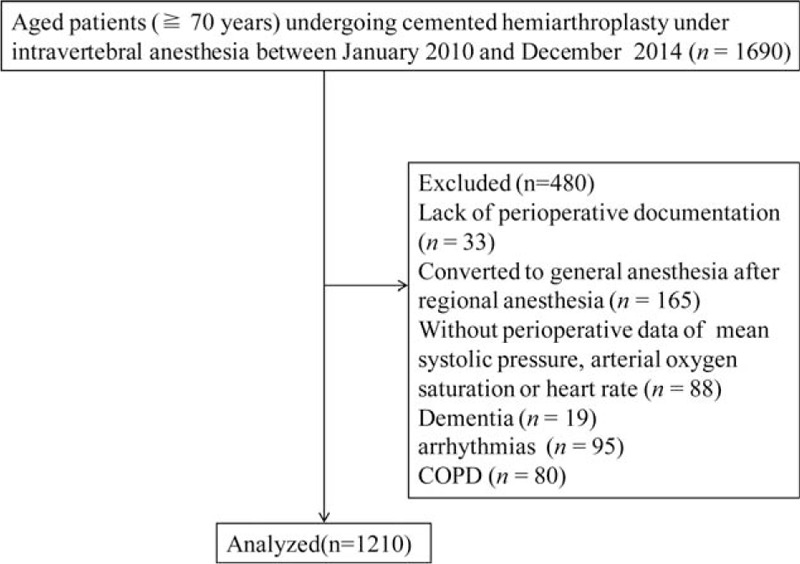
Patient screening and exclusion process.

As illustrated in Fig. 1, 1210 patients were enrolled for review of medical records, but 480 patients met the exclusion criteria.

Baseline characteristics of the remaining1210 patients who had undergone cemented hemiarthroplasty with spinal-epidural anesthesia and single epidural anesthesia for femoral neck fracture are summarized in Table 1. There were no differences in the frequency of intravertebral anesthesias used or the duration of surgery between groups classified according to BCIS grades. After implantation of bone cement, 254 (21.0%) of patients showed a grade 2 of BCIS, and 109 (9.0%) of patients showed a grade 3 of BCIS.

**Table 1:**
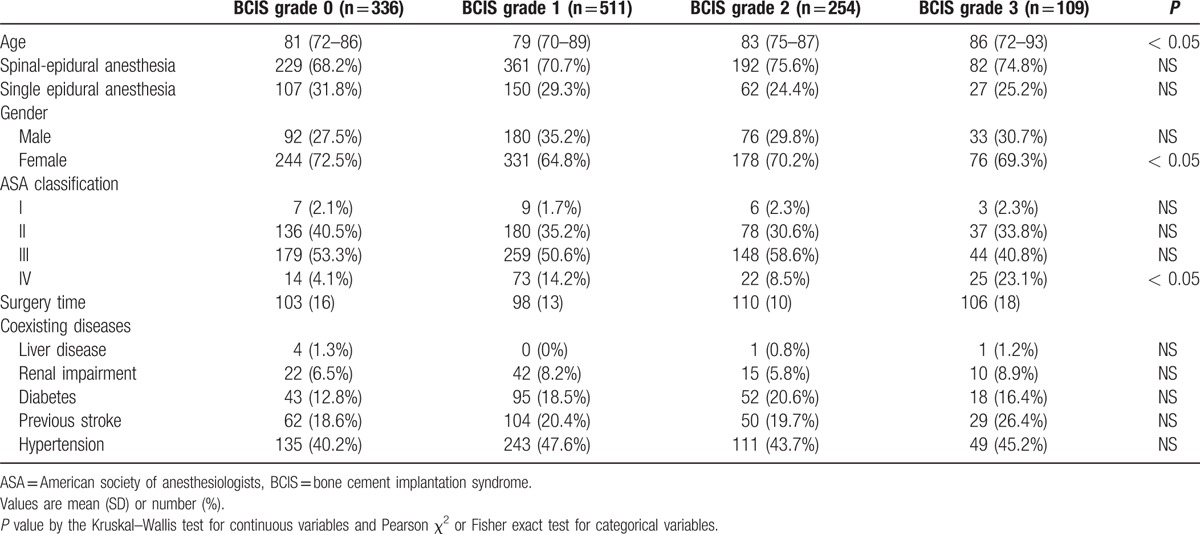
Patient characteristics according to BCIS grades.

Among all included patients, 72.2% (874/1210) showed grade 1 or higher grade of BCIS after cementation. Compared with spinal-epidural anesthesia, single epidural anesthesia showed adjusted odds ratios (95% confidence interval) of 1.25 (1.13–1.43) for grade 1, 1.36 (0.83–2.06) for grade 2, and 3.55 (1.52–7.06) for marked postoperatively grade 3 of BCIS versus grade 0 (Type III *P* *<* 0.0001).

Multivariable analysis showed that single epidural anesthesia was associated with increased odds for elevated BCIS grades compared with spinal-epidural anesthesia after adjusting for other factors (Table 2). Statistically, being a male and surgery time were also identified as factors associated with BCIS grades (Table 2).

**Table 2:**
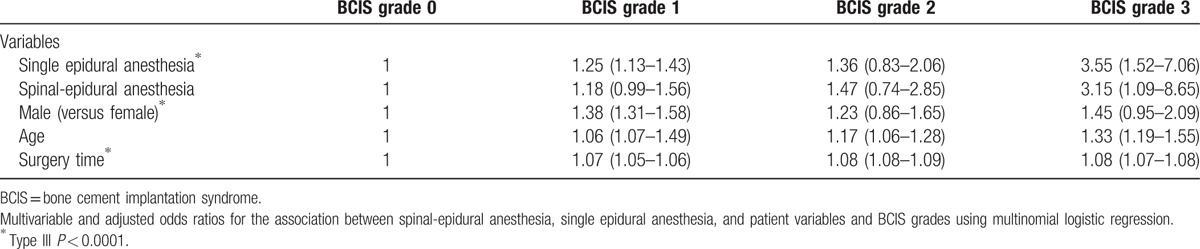
Adjusted odds ratio (95% confidence interval) of BCIS grades.

## 4 Discussion

Present study using the classification system for BCIS to describe the incidence of BCIS and its odds ratio with different ways of intravertebral anesthesia in a single-center population of patients. The main findings were that 72.2% (874/1210) of patients undergoing cemented hemiarthroplasty for hip fracture showed grade 1 or higher grades of BCIS after cementation. Furthermore, compared with spinal-epidural anesthesia, single epidural anesthesia is more associated with the development of the BCIS.

Cemented hemiarthroplasty is an effective method for the reconstruction of the damaged joints caused by various diseases. Because of the risk of osteoporosis, bone cement is routinely used in hemiarthroplasty for hip fracture of elderly patients.^[11,12]^ However, BCIS is a fairly common complication in cemented hemiarthroplasty. Report showed that BCIS is one of the most dangerous complications of hemiarthroplasty.^[13]^ There are about 65% of patients who show BCIS in hemiarthroplasty, which causes about 0.5% to 10% of cardiac arrest and 0.6% to 1% of death.^[10]^

The clinical syndrome of BCIS typically occurs at the time of bone cementation and insertion of the prosthesis. In pathophysiology, pulmonary embolization, release of histamine, and complement activation are 3 main causes of BCIS, which may act to increase pulmonary vascular resistance and then cause ventilation/perfusion disturbances with hypoxia, right ventricular failure, and cardiogenic shock.^[14,15]^

For the elderly patients, the degradation of physical condition will inevitably lead to low tolerance of surgery and anesthesia. It is a challenge for the anesthesiologist to choose an effective and safe way of anesthesia for hemiarthroplasty in elderly patients. In the past, the options included general anesthesia, single spinal anesthesia, and continuous epidural anesthesia. But at present, due to some complications and side effects problems, general anesthesia and single spinal anesthesia are not the preferred method of anesthesia for elderly patients receiving hemiarthroplasty.^[16]^

Epidural anesthesia is an adaptive way for hemiarthroplasty that provides sufficient analgesia and muscle relaxation. However, single epidural anesthesia needs relative larger dose of local anesthetics and exists a certain rate of incomplete nerve block. So the combined spinal-epidural anesthesia looks like a better choice. Combined spinal-epidural anesthesia can not only provide quick analgesia and muscle relaxation, but also reserved the convenience of anesthesia level control.^[17]^ Our results also indicate that spinal-epidural anesthesia is better than single epidural anesthesia for cemented hemiarthroplasty in aged patients. However, the present study is limited by its retrospective nature and the quality of the data in the medical records of our institution.

In summary, according to our data, spinal-epidural anesthesia is a preferred method of anesthesia for cemented hemiarthroplasty in aged patients coexisting respiratory and circulatory instability.

## References

[1] Tsertsvadze A Grove A Freeman K Total hip replacement for the treatment of end stage arthritis of the hip: a systematic review and meta-analysis. *PloS One* 2014; 9:e99804. 25003202 10.1371/journal.pone.0099804 PMC4086719

[2] Muller M Tohtz S Dewey M Age-related appearance of muscle trauma in primary total hip arthroplasty and the benefit of a minimally invasive approach for patients older than 70 years. *Int Orthop* 2011; 35:165–171. 21125270 10.1007/s00264-010-1166-6 PMC3032117

[3] Razuin R Effat O Shahidan MN Bone cement implantation syndrome. *Mal J Pathol* 2013; 35:87–90. 23817399

[4] Newens AF Volz RG Severe hypotension during prosthetic hip surgery with acrylic bone cement. *Anesthesiology* 1972; 36:298–300. 5011424 10.1097/00000542-197203000-00021

[5] Hong CL Liu HP Wu CY Delayed hypoxemia after bone cement insertion during total hip replacement under spinal anesthesia—a case report. *Acta Anaesthesiol Sinica* 2003; 41:47–51. 12747348

[6] Clark DI Ahmed AB Baxendale BR Cardiac output during hemiarthroplasty of the hip. A prospective, controlled trial of cemented and uncemented prostheses. *J Bone Joint Surg Brit* 2001; 83:414–418. 11341430 10.1302/0301-620x.83b3.11477

[7] Sandiford NA Muirhead-Allwood SK Skinner JA Revision of failed hip resurfacing to total hip arthroplasty rapidly relieves pain and improves function in the early post operative period. *J Orthop Surg Res* 2010; 5:88. 21114835 10.1186/1749-799X-5-88 PMC3002320

[8] Parvizi J Holiday AD Ereth MH The Frank Stinchfield Award. Sudden death during primary hip arthroplasty. *Clin Orthop Rel Res* 1999; 369:39–48. 10.1097/00003086-199912000-00005 10611859

[9] Nice EJ Case report: cardiac arrest following use of acrylic bone cement. *Anaesth Intensive Care* 1973; 1:244–245. 4763232 10.1177/0310057X7300100311

[10] Donaldson AJ Thomson HE Harper NJ Bone cement implantation syndrome. *Brit J Anaesth* 2009; 102:12–22. 19059919 10.1093/bja/aen328

[11] Cortes NJ Lloyd JM Koziol L Successful clinical use of daptomycin-impregnated bone cement in two-stage revision hip surgery for prosthetic joint infection. *Ann Pharmacother* 2013; 47:e2. 23324502 10.1345/aph.1R486

[12] He JH Zhou CP Zhou ZK Meta-analysis comparing total hip arthroplasty with hemiarthroplasty in the treatment of displaced femoral neck fractures in patients over 70 years old. *Chin J Traumatol* 2012; 15:195–200. 22863335

[13] Karnwal A Lippmann M Kakazu C Bone cement implantation syndrome affecting operating room personnel. *Brit J Anaesth* 2015; 115:478. 26269477 10.1093/bja/aev280

[14] Griffiths R Parker M Bone cement implantation syndrome and proximal femoral fracture. *Brit J Aanaesth* 2015; 114:6–7. 10.1093/bja/aeu264 25145354

[15] de Froidmont S Bonetti LR Villaverde RV Postmortem findings in bone cement implantation syndrome-related deaths. *Am J Forensic Med Pathol* 2014; 35:206–211. 25072810 10.1097/PAF.0000000000000110

[16] Xue FS Liu GP Sun C Does the method of anesthesia really affect outcomes and survival after total joint replacement? *Canad J Anaesth* 2015; 62:835–836. 25752463 10.1007/s12630-015-0358-3

[17] Hodgson E Combined spinal/epidural anesthesia. *Middle East J Anaesthesiol* 2003; 17:103–112. 12754775

